# Editorial for the Special Issue Research on Biology of Dinoflagellates

**DOI:** 10.3390/microorganisms14051100

**Published:** 2026-05-13

**Authors:** Tania Islas-Flores, Estefanía Morales-Ruiz, Marco A. Villanueva

**Affiliations:** Instituto de Ciencias del Mar y Limnología, Unidad Académica de Sistemas Arrecifales, Universidad Nacional Autónoma de México-UNAM, Prol. Avenida Niños Héroes S/N, Puerto Morelos 77580, QR, Mexico; tania@cmarl.unam.mx (T.I.-F.); esmoru@gmail.com (E.M.-R.)

Protists arose about 1.7 billion years ago and thrived to become a major kingdom on Earth, but around the Triassic period an evolutive explosion occurred which was the advent of the dinoflagellates [1]. The Phylum Dinoflagellata has since then flourished and evolved to reach its current diversity and versatility, as detailed by Soyer-Gobillard in the preamble and introduction to contribution number six of this Issue. Their successful adaptations resulted in their appearance in all kinds of ecological niches, ranging from diverse environments like high-altitude volcanic lagoons to symbiotic societies in freshwater and seawater, forming multi-organismal holobionts. Their success, as with all living organisms, depends on their rapid biological responses to environmental challenges that enable them to react against potential harm or recognize beneficial possibilities that result in adaptation strategies. These microorganisms have evolved to possess a variety of plastid types that have been acquired by secondary and tertiary symbiosis [2]. This has resulted in multiple phenotypic and genotypic traits that enable them to optimize their adaptive responses. Thus, the optimal morphological shapes for inhabiting diverse ecological niches include cysts to allow dormancy in moist land environments like snow or humid soil, flagellated cells to move around in aqueous milieu, and coccoid non-flagellated endosymbiotic cells within a host in marine environments. It is suggested that morphological traits, including the requirements of hydrodynamic streamlining and shear tolerance, evolved to accommodate their swim-based ecology [3]. In addition, their complex physiology and, in many cases, photosynthetic nature, encompasses complex biological processes to respond to environmental cues that include signal-transduction and phototransduction mechanisms in which many players participate to modify protein modulation and gene expression. Advances in the characterization of these microorganisms and efforts towards understanding their physiology and underlying biochemical and molecular mechanisms will allow us to develop strategies to meet critical challenges such as global warming threats on their various aqueous environments, including harmful red tide redistribution [4], phytoplankton primary production, and coral reef survival [5].

The special topic “*Research on Biology of Dinoflagellates*” (online and freely available at https://www.mdpi.com/journal/microorganisms/special_issues/2423M7YS0F, accessed on 6 April 2026) provides an insight into the current research and understanding of the biology of these microorganisms and their interactions at both the cellular and environmental levels. The contributions include (numbered 1–7 according to their online publication date) two comprehensive reviews, with the former covering the most recent findings on Binding Immunoglobulin Proteins (BiP) from Symbiodiniaceae whose chaperone activity switch has been found to respond to light stimuli but are poorly described and characterized in these dinoflagellates, and the latter covering examples of how the inventiveness and innovations in function of various dinoflagellates have rendered them successful protists in the survival-of-the-fittest race. Then, five original research articles on several aspects of dinoflagellate biology and physiology cover a wide variety of subjects which include (a) the analysis of the dynamics of abundance and diversity of proton-pump-rhodopsin dinoflagellate genes during one year in an Atlantic Ocean estuary; (b) study of the influence of allelochemicals from a Chilean strain of *Karenia selliformis* on six representative phytoplankton species; (c) assessment of differential gene expression through transcriptomic analysis of *Heterocapsa bohaiensis* under various nutrient conditions to infer nutrient effects on these dinoflagellates in harmful algal blooms; (d) application of a fluorescent-based method to measure lipid content in dinoflagellates by flow cytometry; and (e) a survey of bacteria and fungi microbiomes associated with cysts of *Scrippsiella acuminata* in different life cycle stages of the dinoflagellate culture. Hereafter, the highlights of the original research articles are presented (in order of online publication appearance).

The work contributed by Zhang et al. highlights a previously neglected light-harvesting mechanism that is an alternative to photosynthesis, namely proton-pump-rhodopsin-based energy phototransduction. They gathered information on the presence of these type of genes in dinoflagellates and isolated six novel full-length proton-pump-rhodopsin (PPR) cDNAs from field seawater dinoflagellates, and two more PPR sequences from cultured *Karlodinium vemeficum*. They then designed specific primers for qPCR and analyzed the dinoflagellate PPR (DiPPR) gene abundance spatially and temporally throughout one year in a temperate estuary of the Atlantic Ocean between Connecticut and Long Island, New York, USA, called the Long Island Sound. The monthly gene abundance of DiPPR varied markedly among stations but was highest in April and, in general, was not influenced by water temperature or nitrogen (N) nutrient concentration. On the other hand, negative correlations of DiPPR gene abundance with salinity and phosphate were found, suggesting that PPR gene expression was upregulated under phosphorus-stressed conditions. None of the 209 dinoflagellate PPR sequences from their DNA samples were identical to the reported DiPPR genes. Their results indicated that some dinoflagellates exist only at certain periods of the year at specific locations, while others are always present. They concluded that the six isolated novel full-length diPPR cDNAs contribute to the DiPPR database and the developed primers which proved to be efficient and specific will be fundamental for future PPR research in natural protist assemblages. The research article by Alfaro-Ahumada et al. focuses on the allelopathic effects of *Karenia selliformis*, one of the major red tide dinoflagellates responsible for fish deaths by toxicity in Chilean coastal waters. They found that allelochemicals from exudates of this dinoflagellate in culture had adverse effects on all target phytoplankton species, including *Phaeodactylum tricornutum*, *Thalassiosira pseudonana*, *Rhodomonas salina*, *Dunaliella tertiolecta*, and two strains of *Heterosigma akashiwo*, one isolated from the Chilean Patagonia and the other from New Zealand. However, high variability was observed in both the strength and nature of the allelopathic impact and, thus, the effects were species-specific on both toxic and non-toxic planktonic species. Cell growth inhibition was observed in *R. salina*, *T. pseudonana*, and the two *H. akashiwo* strains, whereas *D. tertiolecta* and *P. tricornutum* were only slightly affected. Consistently, photosynthetic efficiency was reduced in *R. salina* and *T. pseudonana* but unaffected in *D. tertiolecta*. However, this was not the case for *P. tricornutum*, which showed a marked reduction in photosynthetic efficiency contrasting with the small effect on cell growth. The two *H. akashiwo* strains also exhibited cell growth inhibition when exposed to the exudate. Reactive Oxygen Species (ROS) generation also displayed species-specific effects, even between the two *H. akashiwo* strains, showing increased ROS only in *D. tertiolecta*, *T. pseudonana*, and Chilean *H. akashiwo*, whereas all the other species tested showed no difference with respect to the controls. The authors underscored the importance of studying *K. selliformis* toxicity and its ecological effects on marine ecosystems in Chile to further understand the allelopathic effects of its toxins on phytoplankton ecology and harmful algal bloom dynamics in the Chilean fjords. The article by Peng et al. evaluates the molecular responses of the harmful dinoflagellate *Heterocapsa bohaiensis* to varying N and phosphorus (P) concentrations through transcriptomic analysis. This dinoflagellate, whose metabolic pathways are largely unexplored, forms harmful algal blooms (HABs), causing significant ecological and economic damage in aquaculture ponds, killing crab larvae in culture ponds near Liaodong Bay in the Bohai Sea. Using five different N:P ratios, the authors found that greater deviations from control conditions (880 μM NO^3−^, 32 μM PO_4_^3−^, N:P 27.5) generated a higher number of differentially expressed genes, supporting a pivotal role of nutrient balance in the regulation of this dinoflagellate growth. Gene ontology enrichment analyses revealed that under both N and P limitation, biosynthesis and catabolic processes were upregulated while cell cycle and cell division functions were downregulated, suggesting a shift in resource allocation from proliferation toward cellular survival strategies. KEGG pathway analysis further showed that N limitation led to significant downregulation of photosynthesis and carbon fixation pathways with upregulation of proteasome-related pathways, reflecting the capacity of the dinoflagellate to recycle intracellular N sources from protein degradation. Under P limitation, a notable downregulation of nitrogen metabolism pathways occurred, while ATP synthase genes were upregulated, likely as a compensatory mechanism to sustain energy-intensive cellular processes under P stress. These findings provide valuable insight into the molecular mechanisms by which *H. bohaiensis* adapts to nutrient stress and offer a theoretical basis for the prevention and management of its HABs in aquaculture environments. The next contribution, by Park et al., describes a simple and efficient method to estimate lipid and carbon content in dinoflagellates by fluorescence via flow cytometry. They showed that after BODIPY 505/515 staining, the flow cytometry side scatter effectively represented relative cell size, showing a linear relationship with the equivalent spherical diameter. Larger cells exhibited higher relative lipid content and as the carbon content increased, the lipid content concomitantly increased, but at higher carbon levels a plateau of saturation was observed. The method showed reproducibility and applicability to dinoflagellates since it demonstrated a linear correlation with the sulfo-phospho-vanillin (SPV) method, enabling determination of actual lipid content using FL1 fluorescence and the slope value. Nevertheless, the researchers cautioned about certain parameters that could bias the results; for example, they observed that mixotrophic and benthic species displayed higher lipid content than heterotrophic species due to different nutritional modes and habitats. The last research article, by Yue et al., addresses a notably understudied aspect of dinoflagellate biology—the bacteria–fungi microbiomes associated with different life cycle stages of *Scrippsiella acuminata*, with particular attention to the dormant resting cyst stage. Through 16S and ITS rRNA amplicon sequencing combined with functional prediction via PICRUSt2, the authors characterized both host-attached and free-living microbial communities across vegetative cells and resting cysts under varying temperatures and incubation periods. The results revealed high species diversity in the associated microbiomes, with three bacterial genera, *Stappia*, *Labrenzia*, and *Roseovarius*, identified as core taxa stably co-occurring with *S. acuminata* across all life stages and conditions, likely sustained by their diverse and flexible metabolic capabilities. A clear distinction in the community structure between host-attached and free-living groups was observed, with the former being predominantly copiotrophic and enriched in hydrocarbon-degrading bacteria, while the latter was dominated by oligotrophic taxa adapted to nutrient-scarce conditions. Unexpectedly, the microbiomes associated with resting cysts were significantly enriched in phosphate-solubilizing bacteria and fungi compared to those of vegetative cells. The authors propose that these microorganisms may facilitate P acquisition by the dormant cysts from the surrounding medium, which potentially contributes to the maintenance of cyst viability during dormancy and their subsequent germination. This work defies the conventional view of resting cysts as metabolically inert and opens new lines for investigating the functional roles of phycosphere microbiomes throughout the full dinoflagellate life cycle. Finally, one comprehensive review covers the recent findings on a BiP homolog from Symbiodiniaceae and its phylogenetic relationship with other dinoflagellates, while the other closely examines the evolution and diversity of dinoflagellate inventiveness and adaptation through the lens of cell biology. The review article by Morales-Ruiz et al. provides a synthesis of current knowledge on Binding Immunoglobulin Protein (BiP) homologs in Symbiodiniaceae, a group of dinoflagellates central to coral reef ecosystems. BiP, a member of the HSP70 family resident in the endoplasmic reticulum, is widely recognized for its essential role in protein folding and cellular stress responses; however, its characterization in dinoflagellates remains limited. This review compiles the available sequence data and literature to examine the conservation of BiP at the gene, protein, and structural levels across Symbiodiniaceae and other dinoflagellates, revealing a high degree of evolutionary conservation that suggests preserved functional roles. Notably, the work highlights evidence linking BiP activity to light-responsive regulation, an intriguing feature given the photosynthetic nature of these organisms. At the same time, the authors emphasize the significant gaps that persist, particularly regarding gene regulation, promoter architecture, and functional validation in both free-living and symbiotic stages. By framing BiP within the broader context of stress responses that underpin symbiosis stability, this review underscores its potential importance in coral resilience and calls for further experimental research to elucidate its role in dinoflagellate physiology and coral reef sustainability. And finally, the review article by Soyer-Gobillard, which is dedicated to a pioneer protistologist, Dr. Edouard Chatton, encompasses the evolutionary journey of these remarkable protists from the appearance of their possible primitive ancestors in the Proterozoic era 1500 million years ago to the present. This review can be regarded as a significant dossier of information devoted to describing several representative examples of the various dinoflagellate lifestyles. They include the free-living *Prorocentrum micans* and *Noctiluca scintillans* (autotrophic thecate and heterotrophic athecate, respectively), other “pseudo-noctilucidae”, as well as the thecate *Crypthecodinium cohnii* and the mixotroph *Syndinium* Chatton (parasitic dinoflagellate). Additionally, the different dinoflagellate mitotic systems were compared, and an analysis was carried out on reported observations on the eyespot (ocelloid), an organelle present in the binucleated *Glenodinium foliaceum* Stein and in some Warnowiidae dinoflagellates, which can also be considered an evolutionary marker. She concludes that the diversity and innovations observed in mitosis, meiosis, reproduction, sexuality, cell cycle, locomotion, and nutrition allow us to affirm that dinoflagellates are among the most innovative unicellular organisms in the Kingdom Protista.
